# Impact of Minimal Invasive Robotic Surgery on Recovery From Postoperative Ileus and Postoperative Gastrointestinal Tract Dysfunction

**DOI:** 10.34297/ajbsr.2020.08.001335

**Published:** 2020-05-19

**Authors:** Elvio Mazzotta, Egina Chriseida Villalobos-Hernandez, Alan Harzman, Fievos L Christofi

**Affiliations:** Department of Anesthesiology, The Ohio State University, USA

**Keywords:** Postoperative ileus, POGD, POI, CERAS protocols, GI surgery, robotic surgery, pathogenic mechanisms

## Abstract

Postoperative gastrointestinal tract (GIT) dysfunction (POGD) and postoperative ileus (POI) are common symptoms resulting from small or large bowel surgery associated with extended hospitalizations, increase risk of infections and billions of dollars in health care costs. Open surgery is associated with higher gut surgical trauma / manipulation and worse outcomes compared to minimal invasive surgery. Robotic Surgery may offer added benefit to Colon Enhanced Recovery After Surgery (CERAS) protocols but do not solve the problem. Ultimately, a better understanding of the pathogenic mechanisms of POI and POGD can lead to prophylaxis and enhanced recovery after surgery. The impact of High Pressure Pneumoperitoneum and gut surgical manipulation on GIT dysfunction deserve further investigation.

## Introduction

Postoperative ileus (POI) and Postoperative gastrointestinal dysfunction (POGD) are well-documented complications after abdominal surgery. POI is defined as a temporary impairment of gastrointestinal tract (GIT) motility after surgery, which is associated with significant morbidity (such as oral intolerance, constipation and patient discomfort), and extended hospitalization is estimated to add billions of dollars per year in healthcare costs [1]. The incidence of POI and POGD are unclear and reports range from 3% to 32% and it is higher after open surgery due to more considerable surgical trauma, gut manipulation, extended surgical time, ischemia and inflammation [1,2,3].

The etiology of POI is complex and not well understood and multiple mechanisms may contribute to the pathogenesis of POI, including gut surgical manipulation and trauma, ischemia, sympathetic hyperactivity, inflammation, opioid induced constipation, electrolyte abnormalities, and exacerbation by anesthetic or surgical techniques [2–4].

Furthermore, the incidence and duration of POI correlate with the severity of intestinal inflammation as measured in peritoneal fluid or surgical tissues. It is not surprising then, that open surgery is associated with more inflammation and extended periods of POI than minimally invasive laparoscopic surgery [5,6].

## Minimal Invasive Surgery

In the nineties, surgeons called into question the classic laparotomy and transitioned into a new era of minimally invasive laparoscopic surgery. With the quick development of video computer systems, laparoscopic surgery emerged as an important innovation and shortly was adopted in general surgery [7]. The old dogma that “the bigger the incision, the bigger the surgeon” was later revised to “the smaller the incision the bigger the surgeon” [8]. In parallel with the implementation of minimal invasive surgery the concept of fast track surgery or ‘ERAS’ protocols emerged. Enhance Recovery After Surgery (ERAS) protocols are evidence-based protocols designed to standardize medical care, accelerate patient recovery, attenuate surgical stress response with the final goal of improving outcomes, reduce length of stay, reduce morbidity and lower healthcare costs. Part of the rationale behind the ERAS is to incorporate surgical and anesthesia techniques that can reduce postoperative stress response and improve outcomes [9].

Minimal invasive surgery is one of the pillars of Enhanced Recovery Programs. Over the years with the progression of the technique and equipment, a wide variety of complex procedures were developed and it is now the preferred method of treatment [10]. The obvious benefits of laparoscopic surgery are smaller incisions, limited trauma and reduced inflammation [11,12].

Laparoscopic surgery compared to open surgery is associated with reduced pain, better cosmetic outcomes, earlier recovery of GIT dysfunction, shorter length of stay and reduced cost [13,14]. According to a 2001 Cochrane review, the duration of ileus was 0.9 to 1 days shorter in the laparoscopic group, and contributed to a 1.5 day shorter postoperative hospital stay [15].

A 2016 retrospective study of 509,029 patients undergoing colectomy reported that 52.3% of the cases were open, 46.2% laparoscopic and 1.5% robotic colectomies [16]. Laparoscopic surgery is steadily gaining favor over open surgery, and it is likely this trend will continue. The disadvantages of laparoscopic surgery are a two dimensional view of the abdominal cavity, the limited manual dexterity of surgical instruments, an unstable camera platform, tremor, poor ergonomics and physician fatigue. These limitations are more evident in pelvic surgery compared to some other surgeries due to a confined operating field [17–19].

Robotic laparoscopic surgery is helping tremendously in overcoming some of the pitfalls of conventional laparoscopic surgery. It provides high-quality 3D visualization, wrist-like movement and excellent ergonomics [20]. Due to their unique qualities, the robotic systems have gained popularity in gynecologic and urologic surgery, since the 3D visualization and greater dexterity allow a more precise dissection in the confined pelvis [21].

Since the da Vinci robot gained FDA approval in 2001, there has been an exponential growth in the use of this system. The number of reported systems around the world went from 645 units in 2008 to more than 5000 units in use currently [22].

However, the high cost of the robot and advanced training required, have hampered its more widespread use. There is also a steep learning curve, and as a result, robotic surgery often takes much longer to perform at an obvious cost [17].

Currently there are no head-to-head randomized control trials showing superiority of robotic laparoscopy over conventional laparoscopy in reducing morbidity, POI or POGD, length of stay in the hospital or any long-term consequences to quality of life after discharge. We are still in the early stages of robotic surgery, where the most important thing is the avoidance of open surgery rather than the use of any specific minimally invasive technique [23]. As with other robotic surgeries further refinement in robotic surgery may allow a more precise dissection with less inflammation and potentially decrease complications and shorten hospital stay.

## Mechanical Forces on the GIT during Surgical Procedures

Different types of GIT surgery lead to different mechanical or manipulation forces on the gut and its mesentery. For example, from a surgeon’s perspective, in an open abdominal operation, the small bowel may be moved en masse to one side and held in place with a self-retaining retractor throughout the case, putting direct pressure on the bowel and stretch on its mesentery influencing the nerves and vessels within it. When a particular segment is worked with, it is grasped with the surgeon’s fingers. Depending on the operation, a small part or all of the small and large intestine may require such gross or specific manipulation. Whereas, in a laparoscopic or robotic operation, patient positioning and gravity are used for en masse movement, but the entire intra-abdominal contents are subjected to increased serosal pressure from carbon dioxide insufflation. To manipulate an individual segment of bowel, laparoscopic and robotic instruments use higher pressure over smaller areas than one’s fingers. Compared to laparoscopic surgery, robotic instruments provide a greater range of motion, allowing the surgeon to grasp very specific areas of the bowel or its mesentery, but with instruments capable of generating tremendous pressure between the tips. How all of these forces work together to affect the gut is unknown.

What is clear is that such mechanical forces on the gut do not occur in the normal setting, and in animals, gut surgical manipulation and trauma is used as a model of intestinal inflammation, POI and POGD [24]. Immune cells and inflammatory cells in the gut wall, mucosal epithelial cells, enteric glial cells, neurons and smooth muscle cells among others are implicated in the pathogenic mechanism [2,4,11,24,25,26]. Ongoing studies in our laboratory supported by the National Institute of Diabetes, Digestive and Kidney Diseases (NIDDK) are aimed at testing the novel hypothesis that gut surgical manipulation and trauma causes intestinal inflammation, and induces a ‘reactive enteric glial phenotype’ leading to POI and POGD. In primary human enteric glial cell cultures, inflammation disrupts Ca^2+^ waves, purinergic signaling and mechanosensation in glia [25]. These effects are expected to disrupt motility, since glial Ca^2+^ waves are required for normal motility. The molecular and cellular pathogenic mechanisms of POI are under investigation. Reactive enteric glia are implicated in the pathogenesis of Inflammatory Bowel Diseases, Irritable Bowel Syndrome, constipation and POI as discussed in our recent commentary in [26].

## High versus Low Pressure Pneumoperitoneum

Despite lower incidence of POI with a minimal invasive approach compared with open surgery (laparotomy) [2,27] and improvements seen with implementation of CERAS protocols, this technique requires carbon dioxide (C0_2_) and higher abdominal pressure in order to enhance laparoscopic visualization for surgery. These two variables also contribute to POI and POGD and it can adversely affect the patient’s homeostasis, leading to cardiovascular and respiratory systems changes, decreasing perfusion to abdominal organs and in cardiac preload dependent on blood flow in the inferior vena cava [27,28].

In addition, data in the literature suggest that high pressure pneumoperitoneum (PNP), may have an undesirable effect by causing systemic inflammation and affecting the immune response in the early postoperative period [28]. In order to overcome these negative effects, low CO_2_ PNP pressure has been successfully used to demonstrate its efficacy in reducing postoperative inflammatory response and immune suppression [28].

A recent retrospective chart review study of 400 patients comparing 15mmHg to 12mmHg intra-abdominal pressure (IAP) demonstrated a reduction in POI rates with lower pressure pneumoperitoneum in robotic-assisted radical prostatectomy. Length of stay decreased from 1.76 days to 1.49 days and the rate of POI was reduced to 5% from 12% with low IAP [29]. A prospective randomized study of systemic inflammation and immune response after Laparoscopic Nissen Fundoplication (LNF) performed with standard and low pressure pneumoperitoneum showed that reducing the pressure to 6–8mmHg from 12–14mmHg during LNF reduced the postoperative inflammatory response and avoided postoperative immune suppression/human leukocyte antigen-DR receptor expression [30].

In a preliminary study of robotic prostatectomy comparing 15mmHg versus 12mmHg IAP it was shown that a higher inflammatory response occurs in patients with higher IAP. Circulating IFNγ, TNFα, IL1β and IL4 were higher at 15mmHg than 12mmHg IAP. In contrast, IL10 was higher at lower pressure, whereas IL2, IL12, and IL17 were not changed with pressure. The study suggested that maybe robotic urological surgery at lower pressure is better to reduce the risk of perioperative complications. However, this was only a pilot study and they did not correlate with postoperative complications including POI [31]. We hypothesize that the use of low PNP would reduce systemic inflammation leading to a reduction in the incidence of POI and POGD. To date, no clinical trials have tested whether low pressure pneumoperitoneum is protective against intestinal and systemic inflammation, POI and POGD.

## Conclusion

Despite the implementation of CERAS protocols [32] and minimal invasive techniques, there is still significant POI and POGD associated with prolonged hospitalizations, increased morbidity and health care costs into the billions [32,33]. More randomized control trials are required to evaluate the cost-benefit ratio of robotic surgery versus non-robotic minimal invasive surgery on POI and POGD.

There is evidence to support the hypothesis that high pressure pneumoperitoneum may increase inflammation and the risk of developing POGD. We are currently investigating the impact of mechanical forces such as gut surgical manipulation, trauma and increase in PNP pressure on GI inflammation, POI and POGD. A Clinical Trial is being developed to evaluate the impact of low pressure pneumoperitoneum on POI and POGD.

Overall, a better understanding of the pathogenic mechanisms of POI and POGD is required before we can provide more precise treatment strategies to limit or eliminate these iatrogenic complications [2,3,4].
